# Treosulfan-Based Conditioning Regimen Prior to Allogeneic Stem Cell Transplantation: Long-Term Results From a Phase 2 Clinical Trial

**DOI:** 10.3389/fonc.2021.731478

**Published:** 2021-09-10

**Authors:** Lorenzo Lazzari, Annalisa Ruggeri, Maria Teresa Lupo Stanghellini, Sara Mastaglio, Carlo Messina, Fabio Giglio, Alessandro Lorusso, Tommaso Perini, Simona Piemontese, Magda Marcatti, Francesca Lorentino, Elisabetta Xue, Daniela Clerici, Consuelo Corti, Massimo Bernardi, Andrea Assanelli, Raffaella Greco, Fabio Ciceri, Jacopo Peccatori

**Affiliations:** ^1^Haematology and Bone Marrow Transplant Unit, IRCCS San Raffaele Scientific Institute, Milan, Italy; ^2^Department of Hematology, Oncology and Tumor Immunology, Charité-Universitätsmedizin Berlin, Berlin,Germany; ^3^PhD Program in Public Health, Department of Medicine and Surgery, University of Milano Bicocca, Milan, Italy; ^4^Università Vita-Salute San Raffaele, Milan, Italy

**Keywords:** Treosulfan, ATLG, allogeneic transplant, reduced toxicity, conditioning regimen

## Abstract

**Introduction:**

Reducing toxicities while preserving efficacy in allogeneic stem cell transplant (allo-HCT) remains a particularly challenging problem. Different strategies to enhance the antitumor activity without increasing early and late adverse toxicities of the conditioning regimens have been investigated.

**Methods:**

The aim of “AlloTreo” prospective phase 2 clinical trial was to evaluate the efficacy and safety of a conditioning regimen based on Treosulfan (42 g/m^2^) and fludarabine (https://clinicaltrials.gov/ct2/show/NCT00598624). We enrolled 108 patients with hematological diseases who received a first allo-HCT between June 2005 and January 2011, inside the frame of this trial at our center. Median age at allo-HCT was 49 (21–69) years. Disease Risk Index was low in 14 (13%) patients, intermediate in 73 (67.7%), high in 17 (15.7%), and very high in 4 (3.7%). Donors were human leukocyte antigen (HLA)-matched related in 50 cases, 10/10-matched unrelated in 36, and 9/10-mismatched unrelated in 22. Graft-*versus*-host disease (GvHD) prophylaxis consisted of cyclosporine-A and methotrexate. Anti-T-lymphocyte globulin (ATLG) was administered in patients receiving unrelated allo-HCT. Stem cell source was mainly peripheral blood stem cells (95%).

**Results:**

Conditioning regimen was well tolerated. Full donor chimerism was documented for most patients (88%) at day +30. At 12 years, overall survival (OS) was 41.7% (32.2%–50.9%), progression-free survival (PFS) was 31.7% (23%–40.7%), GvHD-free/relapse-free survival was 20.9% (13.7%–29.1%), cumulative incidence (CI) of relapse was 44.5% (34.9%–53.6%), and transplant-related mortality (TRM) was 22.5% (15.1%–30.9%). CI of acute GvHD grades II–IV was 27.8% (19.7%–36.5%) at 100 days; 12-year CI of chronic GvHD was 40.7% (31.3%–49.9%). Relevant long-term adverse effects were 10 secondary malignancy, 3 fatal cardiovascular events, and 1 late-onset transplant-associated thrombotic microangiopathy. Ten successful pregnancies were reported after allo-HCT. In multivariate analysis, older age (≥60 years) at transplant [hazard ratio (HR), 2.157; p = 0.004] and a high/very high disease risk index (HR, 1.913; p = 0.026) were significantly associated with a lower OS.

**Conclusions:**

Overall, our data confirmed the myeloablative potential and safe toxicity profile of full dose Treo (42 g/m^2^) especially for the younger population.

## Introduction

Allogeneic hematopoietic stem cell transplantation (allo-HCT) is an increasingly offered treatment option for the management of hematological malignancies (1). Transplant-related mortality (TRM) has fallen in the past 40 years; however, the major causes of treatment failure remain disease relapse and treatment toxicities (2). The use of reduced intensity conditioning regimens (RICs) coupled with expansion of alternative donor stem cell sources has dramatically increased the number of patients who can benefit from allo-HCT. The introduction into clinical practice of less toxic chemotherapeutic agents, new antimicrobials, and more effective graft-*versus*-host disease (GvHD) treatments has significantly reduced TRM over the last decades (3). In this context, optimization of both patient selection and conditioning regimen is critical to improve outcomes.

The conditioning regimen given prior to allo-HCT has the aim of suppressing host immunity, allowing donor cell engraftment, and ablating the underlying malignancy. Bacigalupo et al. (4) have classified the intensity of conditioning regimens into myeloablative (MAC), RIC, and non-myeloablative based on the expected duration and reversibility of cytopenias. Even if more effective against disease relapse, MAC regimens also enhance toxicities, further limiting the overall outcome of allo-HCT. Reduced-toxicity conditioning (RTC) regimens, based on the use of fludarabine and an alkylating agent, have been designed to allow a safer administration of dose-intensive myeloablative therapy (5, 6). This area of investigation will likely continue to be of interest in terms of optimizing transplant results.

Treosulfan (Treo), a water-soluble bifunctional alkylating drug, demonstrated an advantageous toxicity profile over standard conditioning regimens in preliminary experiences (7, 8). It has been increasingly applied to pediatric and adult patients with hematological malignancies, showing low risk of organ toxicity and treatment-related mortality combined with effective immunosuppressive and cytotoxic properties (9–14). In a recent multicenter randomized phase 3 trial, Treo demonstrated non-inferiority over busulfan (Bu) when used in combination with fludarabine in patients with advanced age or comorbidities, suggesting a potential to become a standard preparative regimen in this population (15).

The aim of the “AlloTreo” study—a prospective, multicenter, non-randomized, open-label, phase 2 clinical trial—was to evaluate the efficacy and safety of Treo in combination with fludarabine as a preparative regimen for allo-HCT. Herein, we report the long-term outcomes for this study population.

## Materials and Methods

This is a long-term, single-center, retrospective analysis of prospectively collected data from the clinical phase 2 multicentric trial “AlloTreo.” Primary endpoint of this trial was neutrophil engraftment and the incidence of CTC-AE grade 3 and 4 adverse events. Secondary endpoints were the evaluation of overall survival (OS), progression-free survival (PFS), TRM, cumulative incidence (CI) of relapse/progression, CI of acute GvHD (aGvHD) and chronic GvHD (cGvHD), bone marrow donor chimerism at +28 and +100, and the incidence of Epstein–Barr virus (EBV) reactivations. The study protocol was approved by the institutional review board of San Raffaele Scientific Institute and complied with our country-specific regulatory requirements. The study was conducted in accordance with the Declaration of Helsinki and good clinical practice guidelines. Written informed consent was provided by all patients. Inclusion and exclusion criteria of the trial are provided in **Table S1**.

We included in this long-term analysis those patients who received an allo-HCT at our center from a matched-related or unrelated donor using peripheral blood stem cell (PBSC) or bone marrow (BM) as a source. Overall, we included in this study 108 patients with hematological diseases—99 enrolled in the “AlloTreo” study and 9 inside a pilot project performed before starting with the trial—who received a first allo-HCT at San Raffaele Scientific Institute in Milan between June 2005 and January 2011. The last follow-up was January 1, 2021.

The RTC regimen consisted of Treo 14 g/m^2^ daily for 3 days (from day −6 to −4) and fludarabine 30 mg/m^2^ for 5 days (from day −6 to −2) (**Figure S1**). GvHD prophylaxis consisted of cyclosporine-A (CSA) from day −1 and methotrexate 15 mg/m^2^ on day +1, with consecutive doses of 10 mg/m^2^ given on days +3 and +6. Anti T-lymphocyte globulin (ATLG, Neovii) was given as part of the conditioning regimen (10 mg/kg from day −4 to −2) to patients receiving grafts from an unrelated donor. A single dose of Rituximab 500 mg was added in these cases considering the high risk of posttransplant lymphoproliferative disorders related to the *in vivo* T-cell depletion. Supportive care and antimicrobial prophylaxis followed institutional guidelines.

### Study Definitions

Complete remission was defined in case of absence of disease activity by BM evaluation or imaging, according to the underlying disease. All patients not falling within this definition were categorized as having active disease. Additionally, patients were stratified by status at the time of transplantation according to the disease risk index (DRI) defined by Armand et al. (16). Comorbidities at time of transplantation were evaluated according to the hematopoietic cell transplantation-specific comorbidity index (HCT-CI) (17). Human leukocyte antigen (HLA) compatibility among donor–recipient pairs was assessed by 10 loci molecular typing (HLA-A, HLA-B, HLA-C, HLA-DRB1, and HLA-DQB1) at the allelic level.

Neutrophil engraftment was defined as the first of 3 consecutive days with neutrophil counts ≥0.5 ×10^9^/L after transplantation, and platelet engraftment was defined as platelet counts ≥20 ×10^9^/L in the absence of growth factors or transfusions during the preceding 7 days. Disease follow-up during posttransplant period consisted of BM evaluations carried out monthly for the first 3 months and then two times a year for the first 5 years. Donor-recipient chimerism was assessed on unfractionated BM aspirate samples with a commercial assay based on short-tandem repeats analysis (AmpFISTR Profiler Plus PCR Kit; Applied Biosystem, Carlsbad, CA). Patients were considered fully chimeric if their unfractionated BM samples were ≥95% donor.

Clinical diagnosis and grading of aGvHD were made according the Glucksberg criteria (18), while cGvHD diagnosis and grading were based on the National Institutes of Health consensus criteria (19). GvHD was treated per institutional protocols considering the European Society for Blood and Marrow Transplantation recommendations. Tapering of GvHD prophylaxis occurred as per protocol, in the absence of GvHD signs, starting from day +90 after allo-HCT, and definitively withdrawn at day +180. Cytomegalovirus (CMV) and EBV were monitored at least weekly until day +100 in peripheral blood plasma samples.

### Statistical Analysis

Primary endpoint of our retrospective analysis was OS. Secondary endpoints were PFS, TRM, incidence of neutrophils and platelets engraftment, CI of relapse/progression (RI), and CI of acute and chronic GvHD. OS was defined as the time from transplant to death from all causes. PFS was defined as the time to death or relapse/progression, whichever came first. TRM was defined as death without evidence of relapse. Competing risks were as follows: death without engraftment for engraftment, death without relapse for RI, relapse for TRM, and death without GvHD for aGvHD and cGvHD.

Main clinical characteristics were studied for associations with outcomes by univariate analysis using the log-rank test for PFS, OS, and GvHD-free/relapse-free survival (GRFS), while Grey’s test was employed for CI of aGvHD, cGvHD, RI, and TRM. A 95% confidence interval (95% CI) was considered. A p-value lower than 0.05 was interpreted as significant.

Multivariate analysis was performed using the Cox proportional-hazard model. All factors known to influence outcome and factors associated with a univariate analysis p < 0.10 were first included in the model. Subsequently, a stepwise backward procedure was used with a cutoff significance level of 0.10 for deleting factors from the model. The type I error rate was fixed at 0.05 for determination of factors associated with time to event.

Analyses were performed using SPSS version 25.0 (IBM Corporation, Armonk, NY) and R statistical software version 4.0.4 (R Development Core Team, Vienna, Austria).

## Results

Patients’ and transplant characteristics are provided in **Table 1**. Acute myeloid leukemia (AML) was the most common disease with 36 cases (33.3%), followed by non-Hodgkin’s lymphoma (NHL; n = 21, 19.5%) and myelodysplastic syndrome (MDS; n = 15, 13.9%). Donor types were as follows: matched related donor (MRD) in 50 cases, 10/10-matched unrelated donor (MUD) in 36, and 9/10-mismatched unrelated donor (MMUD) in 22 patients. The source of stem cells mainly consisted in unmanipulated PBSC (95%); only five patients underwent a BM allo-HCT. Almost half of the patients (45%) were transplanted in complete remission: 37 in first complete remission (CR1) and 12 in second or subsequent complete remission (CR ≥ 2). Disease status at the time of allo-HCT for the remaining 55 patients was partial remission (PR) in 18 (16.7%) cases and active/advanced disease (AD) in 37 (34.3%), 19 (17.6%) of which, mainly suffering from myelodysplastic syndrome or primary myelofibrosis, received an upfront allo-HCT. DRI was low/intermediate in 87 (80.6%) patients and high/very high in 21 (19.4%). Median HCT-CI was 1 (range, 0–7): 0–1 in 57 (52.8%) patients, 2–3 in 35 (32.4%), and ≥4 in 16 (14.8%) cases. Median follow-up was 148 (range, 58–189) months.

**Table 1:** Patients’ and transplant characteristics.

		**TOTAL (*N*=108)**
**Patient age years, median (range)**		49 (21-69)
**Patient sex, male (%)**		76 (70)
**HCT-CI, median (range)**		1 (0-7)
**Diagnosis, *n* (%)**	AML	36 (33.3)
	ALL	11 (10.2)
	MPAL	1 (0.9)
	MDS	15 (13.9)
	CML	1 (0.9)
	MPD	7 (6.5)
	MDS/MPN	2 (1.9)
	MM	5 (4.6)
	HL	4 (3.7)
	NHL	21 (19.4)
	CLL	4 (3.7)
	Other	1 (0.9)
**Disease status at transplant, *n* (%)**	CR1	40 (37)
	CR≥2	13 (12)
	PR	18 (16.7)
	Relapse/PD	18 (16.7)
	Upfront	19 (17.6)
**DRI, *n* (%)**	Low	14 (13)
	Intermediate	73 (67.6)
	High	17 (15.7)
	Very high	4 (3.7)
**Type of donor, *n* (%)**	MRD	50 (46.3)
	MUD	36 (33.3)
	MMUD	22 (20.4)
**ATLG, *n* (%)**		58 (53.7)
**Stem cell source, *n* (%)**	PBSC	103 (95.4)
	BM	
**PBSC graft content, median (range)**	CD45+ cells x10^8^/Kg	8.7 (1.3-25.5)
	CD34+ cells x10^6^/Kg	7.0 (2.6-17.8)
	CD3+ cells x10^8^/Kg	3.0 (0.6-9.9)
**BM graft content, median (range)**	CD45+ cells x10^8^/Kg	3.1 (2.6-8.5)
	CD34+ cells x10^6^/Kg	4.0 (2.4-7.0)
	CD3+ cells x10^6^/Kg	0.8 (0.3-12.2)
**H/D CMV status, *n* (%)**	pos/pos	62 (57.5)
	pos/neg	31 (28.7)
	neg/pos	6 (5.5)
	neg/neg	9 (8.3)

HCT-CI, Hematopoietic cell transplantation-comorbidity index; AML, acute myeloid leukemia; ALL, acute lymphoblastic leukemia; MPAL, mixed phenotype acute leukemia; MDS, myelodysplastic syndromes; CML, chronic myeloid leukemia; MPN, myeloproliferative neoplasm; MDS/MPN, myelodysplastic-myeloproliferative neoplasms; MM, multiple myeloma; HL, Hodgkin lymphoma; NHL, non-Hodgkin lymphoma; CLL, chronic lymphocytic leukemia; CR1, first complete remission; CR2, second complete remission; PR, partial remission; PD, progressive disease; DRI, Disease Risk Index; MRD, matched related donor; MUD, matched unrelated donor; MMUD, mismatched unrelated donor; ATLG, anti T-lymphocyte globulin; PBSC, peripheral blood stem cells; BM, bone marrow; H/D, host/donor; CMV, cytomegalovirus.

### OS, PSF, and GRFS

**Figure 1:**
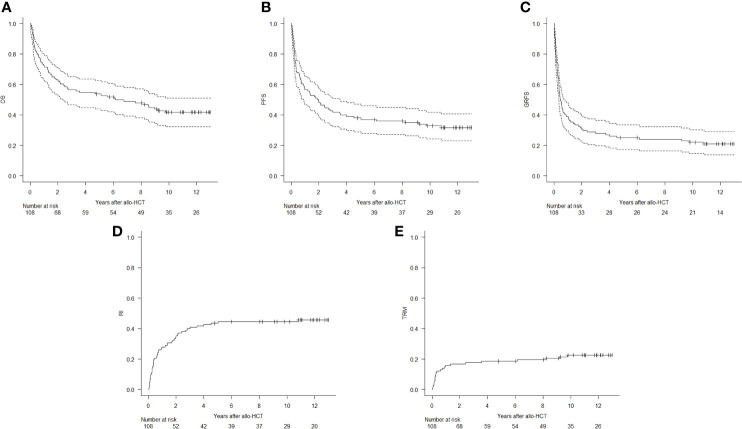
Kaplan–Meier estimates of overall survival (OS, **A**), progression-free survival (PFS, **B**), and graft-*versus*-host-free/relapse-free survival (GRFS, **C**), and cumulative incidence of relapse/progression (RI, **D**) and transplant-related mortality (TRM, **E**).

At 12 years, OS was 41.7% (95% CI, 32.2%–50.9%), PFS was 31.7% (95% CI, 23%–40.7%), and GRFS was 20.9% (95% CI, 13.7%–29.1%) (**Figure 1**).

Results of the univariate analysis are reported in **Table 2**. In multivariate analysis, risk factors for a lower OS were age ≥60 years and having a high/very high DRI. Transplantation using a MUD and a high/very high DRI were independently associated with lower PFS (**Table 3**).

**Table 2:** Univariate analysis for significant transplant outcomes at 12 years.

Variables	Median (95% CI)				
	OS	PFS	GRFS	TRM	RI
**Recipient age**					
**<60 years**	50.1% (38.7–60.5)	39.3% (28.7–49.8)	24.6% (15.8–34.4)	16.2% (9.2–25.1)	44.4% (33.3–54.9)
**≥60 years**	16.5% (5.3–33)	6.7% (0.6–23.7)	6.9% (0.7–24.3)	41.6% (22.1–60)	51.1% (27.3–70.7)
	***p = 0.001***	***p = 0.019***	*p = 0.199*	***p = 0.004***	*p = 0.916*
**DRI**					
**Low/intermediate**	46.1% (35.2–56.4)	38.7% (28.4–48.8)	25.1% (16.5–24.6)	22.1% (14–31.5)	39.1% (28.8–49.3)
**High/very high**	23.8% (8.7–43.1)	4.8% (0.3–19.7)	4.8% (0.3–19.7)	23.8% (8.2–43.9)	71.4% (44–87.1)
	***p = 0.015***	***p < 0.001***	***p = 0.008***	*p = 0.986*	***p = 0.002***
**Donor type**					
**MRD**	48.5% (33.8–61.8)	43.1% (28.9–56.4)	21.7% (11.5–33.9)	14.8% (6.4–26.6)	42% (28.1–55.3)
**MUD**	32.7% (18.2–48.2)	11.9% (3.6–25.7)	12.2% (3.7–26)	30.8% (16.4–46.3)	57.1% (37.9–72.4)
**MMUD**	40.9% (20.9–60.1)	36.4% (17.4–55.7)	31.8% (14.2–51.1)	27.3% (10.7–47)	36.4% (16.8–56.3)
	*p = 0.161*	***p = 0.016***	*p = 0.331*	*p = 0.097*	*p = 0.373*
**ATLG**					
**Yes**	35.8% (23.7–48)	21.8% (12.2–33.3)	21.7% (11.5–33.9)	29.4% (18.2–41.5)	48.6% (35–61)
**No**	48.5% (33.8–61.8)	43.1% (28.9–56.4)	20.3% (11–31.5)	14.8% (6.4–26.6)	42% (28.1–55.3)
	*p = 0.073*	***p = 0.02***	*p = 0.537*	***p = 0.031***	*p = 0.731*

Outcomes were not statistically different if patients were grouped according to HCT-CI score, host/donor CMV mismatch, or stem cell source (not shown).

CI, confidence interval; OS, overall survival; PFS, progression-free survival; GRFS, graft-versus-host-free/relapse-free survival; TRM, transplant-related mortality; RI, relapse incidence; DRI, disease risk index; MRD, matched related donor; MUD, matched unrelated donor; MMUD, 9/10-mismatched unrelated donor; ATLG, anti T-lymphocyte globulin.

Bold values were statistically significant.

**Table 3:** Multivariate analysis for main outcomes.

	HR	95%CI per HR		p-value
		Lower	Upper	
***OS***				
**Age ≥60 *vs*. <60**	**2.157**	**1.286**	**3.616**	**0.004**
**HCT-CI ≥3 *vs*. <3**	1.233	0.733	2.076	0.430
**DRI H/VH *vs*. L/I**	**1.913**	**1.081**	**3.386**	**0.026**
**MMUD *vs*. MRD**	1.253	0.636	2.470	0.515
**MUD *vs*. MRD**	1.543	0.875	2.722	0.134
**ATLG *vs*. no ATLG**	1.205	0.606	2.395	0.594
***PFS***				
**Age ≥60 *vs*. <60**	1.588	0.972	2.596	0.065
**HCT-CI ≥3 *vs*. <3**	1.242	0.764	2.019	0.383
**DRI H/VH *vs*. L/I**	**2.304**	**1.351**	**3.930**	**0.002**
**MMUD *vs*. MRD**	1.335	0.704	2.530	0.376
**MUD *vs*. MRD**	**1.880**	**1.117**	**3.167**	**0.018**
**ATLG *vs*. no ATLG**	1.281	0.671	2.447	0.453
***TRM***				
**Age ≥60 *vs*. <60**	**3.072**	**1.381**	**6.831**	**0.006**
**HCT-CI ≥3 *vs*. <3**	1.449	0.647	3.245	0.367
**DRI H/VH *vs*. L/I**	1.209	0.445	3.285	0.709
**MMUD *vs*. MRD**	1.398	0.528	3.700	0.500
**MUD *vs*. MRD**	1.734	0.585	5.142	0.321
**ATLG *vs*. no ATLG**	2.230	0.922	5.394	0.075
***RI***				
**Age ≥60 *vs*. <60**	1.115	0.586	2.122	0.741
**HCT-CI ≥3 *vs*. <3**	1.072	0.581	1.979	0.824
**DRI H/VH *vs*. L/I**	**3.086**	**1.664**	**5.724**	**<0.001**
**MMUD *vs*. MRD**	1.061	0.466	2.415	0.889
**MUD *vs*. MRD**	1.607	0.865	2.984	0.133
**ATLG *vs*. no ATLG**	1.029	0.446	2.378	0.946

CI, confidence interval; HR, hazard ratio; OS, overall survival; PFS, progression-free survival; TRM, transplant-related mortality; RI, relapse incidence; HCT-CI, hematopoietic cell transplantation-specific comorbidity index; DRI, disease risk index; H, high; VH, very high; L, low; I, intermediate; MMUD, 9/10-mismatched unrelated donor; MUD, 10/10-matched unrelated donor; ATLG, anti T-lymphocyte globulin.

Bold values were statistically significant.

### Toxicity, Viral Infections, and Fertility

Conditioning regimen was well tolerated: non-hematological adverse events mainly consisted of low grade (CTC-AE grades 1 and 2) gastrointestinal mucositis and skin rash. Two patients died due to infectious complications before neutrophil engraftment. No other CTC-AE grade 3 or 4 conditioning-related adverse events occurred. Hepatic sinusoidal obstruction syndrome (SOS) cases were not documented.

CMV reactivations occurred in 63 (58.3%) patients. Five cases developed a CMV disease and were treated according to institutional guidelines. EBV reactivation occurred in three (2.8%) patients, prompting the need for Rituximab treatment in two of them according to institutional guidelines. No cases of post-transplant lymphoproliferative disease were documented.

Ten (22%) of our 45 long-term survivors were diagnosed with a second cancer during follow-up. Median time from allo-HCT to diagnosis of the secondary solid tumor was 7.9 (1.3–11) years. Second cancer types were as follows: three non-melanoma skin cancer (one basal and two squamous cell carcinomas), one renal neoplasia, one metastatic colon cancer, four pulmonary neoplasms, and one gastric tumor. Four of these patients were previously diagnosed with cGvHD and received an immunosuppressive treatment before the development of the second malignancy. Three of these patients died due to cancer-related complications. Two women were diagnosed with a human papillomavirus-related cervical intraepithelial neoplasia after allo-HCT. No secondary hematological malignancies were observed during the follow-up.

Other toxicities diagnosed during long-term follow-up in long-term survivors were hypothyroidism (five cases), acute myocardial infarction (two cases), ischemic stroke (two cases), heart failure associated with chronic atrial fibrillation (one case), cutaneous herpes zoster (five cases), postherpetic neuralgia (two cases), secondary hemosiderosis treated with deferasirox or phlebotomy (six cases), and avascular necrosis of the femoral head (four cases).

At last follow-up, 10 successful pregnancies were reported after allo-HCT by 6 patients (5 men and 1 woman). Median age at transplant of this group was 31 (range, 25–41) years. The pregnancies resulted in successful deliveries of 10 live-born singletons (3 boys and 7 girls). Pregnancy outcome was uncomplicated in all cases, and there was no delivery-related complication in the mothers. Seven pregnancies were achieved with spontaneous conception, while two men reported use of cryopreserved sperm (one case unknown). The only woman that successfully carried a pregnancy to term was transplanted from her HLA-identical sister at the age of 28 for an intermediate risk AML in CR1. During the posttransplant period, she was diagnosed with moderate cGvHD at day +192 managed with topical therapy and immunosuppression adjustments; CSA was definitively suspended after 9 months from diagnosis. Pregnancy was reported after 7 years from the date of allo-HCT. No sign of disease recurrence or GvHD flare-up was documented at last follow-up.

### Engraftment and GvHD

The CI of engraftment was 96% at day +28 for neutrophils and 95% at day +100 for platelets. Median time to neutrophil and platelet engraftment was 16 (range, 11–39) days and 14 (range, 8–47) days, respectively. No primary or secondary graft failure was observed. Day +28 chimerism was evaluable in 101 patients, 95 of which displayed a ≥95% donor chimerism. Three more patients converted to full donor chimerism at the evaluation of day +100.

**Figure 2:**
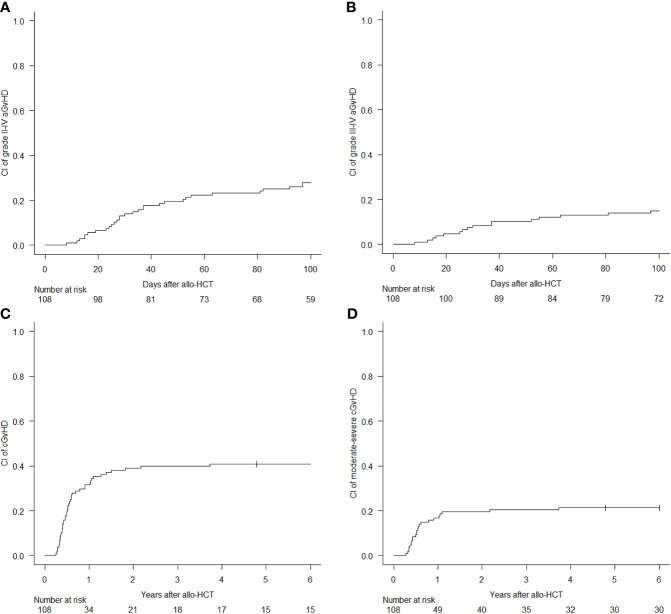
Cumulative incidence (CI) of acute graft-*versus*-host disease (GvHD) grade II–IV **(A)** and III-IV **(B)**, and CI of chronic GvHD all grades **(C)** and moderate-to-severe **(D)**.

**Figure 3:**
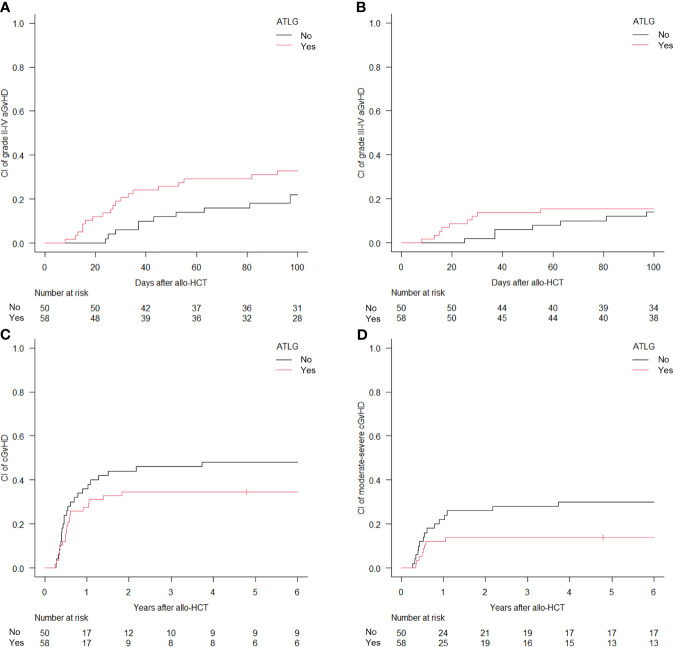
Cumulative incidence (CI) of acute graft-*versus*-host disease (GvHD) grade II–IV **(A)** and III–IV **(B)** and CI of chronic GvHD all grades **(C)** and moderate-to-severe **(D)** according to anti T-lymphocyte globulin administration.

The 100-day CI of aGvHD grade II–IV and grade III–IV was 27.8% (95% CI, 19.7%–36.5%) and 14.8% (95% CI, 8.9%–22.2%), respectively (**Figure 2**). Median time to development of aGvHD was 68 days. Skin was the most frequent organ affected by aGvHD: 30 cases displayed an isolated cutaneous form, while other 16 cases developed a skin involvement in association with a visceral one (6 with lower gastrointestinal and 10 with liver disease). Acute GvHD with isolated visceral organ involvement was diagnosed in 10 cases (2 lower gastrointestinal and 8 liver disease). In univariate analysis, the administration of ATLG did not show a significant difference in the CI of aGvHD grade II–IV [32.8% (95% CI, 21%–45%) for the ATLG group *versus* 22% (95% CI, 11.7%–34.4%); p = 0.432] and grade III–IV [15.5% (95% CI, 7.6%–26%) for the ATLG group *versus* 14% (95% CI, 6.1%–25.1%); p = 0.61] (**Figure 3**). Similarly, disease status at transplant and donor type had no impact for these two outcomes.

**Figure 4:**
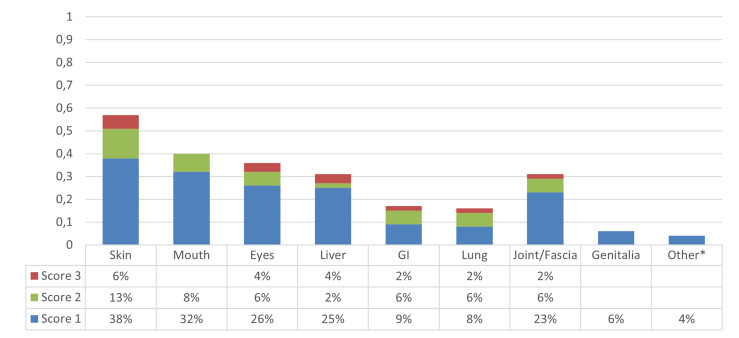
Graphic representation of the distribution of organs involved by chronic graft-*versus*-host disease (cGvHD) in the overall population. *Renal cGvHD.

At 6 years, the CI of cGvHD and moderate-to-severe cGvHD was 40.7% (95% CI, 31.3%–49.9%) and 21.3% (95% CI, 14.1%–29.5%), respectively (**Figure 2**). Median time to cGvHD occurrence was 267 days. In univariate analysis, ATLG administration was associated with a significant reduction in the 6-year CI of moderate-to-severe cGvHD [13.8% (95% CI, 6.4%–24.1%) for the ATLG group *versus* 30% (95% CI, 17.9%–43.1%); p = 0.0475], while there was no difference in the CI of cGvHD all grades [34.5% (95% CI, 22.4%–46.9%) for the ATLG group *versus* 48% (95% CI, 33.4%–61.2%); p = 0.178] (**Figure 3**). Median length of CSA administration was 220 (range, 25–1,966) days. Overall, 25 (15 MRD, 6 MUD, and 4 MMUD) patients developed a moderate-to-severe cGvHD requiring a prolonged systemic immunosuppressive therapy. Organ involvements of patients diagnosed with cGvHD are displayed in **Figure 4** and **Figure S2**. At the time of last follow-up, 41 of 47 patients were alive and off their assigned immunosuppression.

### Relapse and TRM

RI was 44.5% (95% CI, 34.9%–53.6%) at 12 years (**Figure 1**). Median time for relapse occurrence was 8 (range, 0.4–131.5) months. Overall, forty-nine (45%) patients died from disease relapse/progression. There was no difference in the 12-year RI between the four more frequent disease types in our cohort [44% (95% CI, 27.6%–60%) for AML, 36.4% (95% CI, 10%–64.2%) for acute lymphoblastic leukemia, 35% (95% CI, 11.1%–60.6%) for MDS, and 42.9% (95% CI, 21.1%–63.1%) for NHL; p = 0.865].

Twenty patients received a second allo-HCT for disease relapse after a median of 256 (range, 28–1,870) days from the first allo-HCT. In 11 cases a different donor was chosen: 10 patients underwent a haploidentical family donor allo-HCT and 1 patient a cord blood transplant. As for the other nine patients, we made use of the cryopreserved PBSC from the first donor. Treo in association with a purine nucleoside analog (fludarabine or clofarabine) was the most frequent conditioning regimen used (10 cases), while an intensification with 4 Gy of total-body irradiation was used in 4 cases. At last follow-up, 3 out of these 20 patients are alive and in complete remission; 11 died from disease progression and 6 for causes related to the second allogeneic procedure. One patient underwent a third allo-HCT using a haploidentical family donor and a MAC regimen to treat a further disease relapse diagnosed 2 years after the second transplant: she is alive and in complete remission 6 years after the third allo-HCT.

CI of TRM was 10.2% at 100 days (95% CI, 5.4%–16.8%) and 22.5% (95% CI, 15.1%–30.9%) at 12 years (**Figure 1**). Twenty-five patients died from transplant-related causes: six from infections (three sepsis and three pneumonias), three from GvHD, three from multiorgan failure, one from arrhythmia, one from stroke, one from acute myocardial infarction, one from late-onset transplant-associated thrombotic microangiopathy, three from secondary malignancies, and six for unknown causes.

Results of the univariate analysis are reported in **Table 2**. In multivariate analysis, age ≥60 years was independently associated with a higher risk of TRM. High/very high DRI was a risk factor for higher RI (**Table 3**).

## Discussion

Allo-HCT conditioning regimen has rapidly changed from a one regimen that fits all to multiple potential regimens tailored on disease characteristics and patient comorbidities. In this setting, even in advanced disease stages, Treo has increasingly been employed owing to its low risk of organ toxicity and TRM (7, 15, 20–24).

At the time of this phase 2 clinical trial accrual, Treo was approved only for the treatment of advanced ovarian carcinoma. Treo exhibited low inter- and intrapatient variability in pharmacokinetic studies; gastrointestinal mucositis, skin toxicity, and metabolic acidosis were reported as dose-limiting adverse effects (25, 26). Preliminary clinical trials in the allo-HCT setting demonstrated an advantageous toxicity profile of Treo up to a dose of 42 g/m^2^ when compared with other standard conditioning regimens (7, 8). Thereafter, Treo was approved by the European Medicines Agency (EMA) at a total dose of 30 g/m^2^ according to the results of a multicenter randomized phase 3 trial in older and comorbid patients.^15^

Although potentially limited by the presence of single-center data, our long-term analysis confirms that a full-dose Treo-based conditioning regimen displays a strong myeloablative and immunosuppressive potential coupled with a good safety profile, in line with other recent studies (9, 15, 20–23, 27–29). A fast and stable full donor engraftment was achieved by most of our patients, toxicities were limited, and no case of SOS was reported. Importantly, in our series, 20 patients were able to proceed to a second allo-HCT for the treatment of disease relapse, a further proof of the low cumulative toxicity of this conditioning combination. We were able to report detailed long-term adverse events in our population: 10 patients were diagnosed with a secondary malignancy, 3 patients died from cardiovascular diseases, 1 patient died from a late-onset transplant-associated thrombotic microangiopathy. Six long-term survivors from our study were able to achieve a successful pregnancy or fatherhood after allo-HCT, underlining the lower gonadal toxicity of Treo as compared with other alkylating agents such as Bu (30, 31).

The results of our study are in line with those regarding Bu-based conditioning regimens (15, 20, 21, 23, 32, 33). The immunosuppressive activity of Treo facilitates stem cell engraftment, making it an attractive candidate for this clinical context (21, 27, 34).

In our study, older age at transplant was associated with a lower OS and an increased TRM. Age is a known factor associated with TRM due to the burden of comorbidities and frailty. Indeed, in our series, the use of Treo at a daily dose of 14 g/m^2^ may in part explain this finding. Considering that RI was similar between the two age groups, reducing the Treo dose could possibly improve allo-HCT outcomes in the older population, as also confirmed in other trials (5, 15, 23).

Disease recurrence was a major issue in our study, especially for patients with a high/very high DRI. Intensification of the conditioning regimen with the addition of a second alkylating agent or total body irradiation based on the underlying disease type could be implemented in this group of patients to counteract posttransplant disease relapse. Furthermore, owing to the fast and stable recovery provided with this conditioning protocol, the use of preemptive maintenance therapies in the early posttransplant period may be explored in these high-risk patients (35). Moreover, an additional limitation to our study, mainly related to its long-standing enrollment phase, is the absence of detailed data on measurable residual disease, a rich field of research where many advances have been made in recent years (36).

The use of *in vivo* T-cell depletion with ATLG for patients undergoing unrelated donor allo-HCT was able to significantly reduce the incidence of moderate-to-severe cGvHD at the expense of a higher risk of TRM and worse PFS, with a trend towards a lower OS in univariate analysis. At the time of this trial, T-cell depletion with ATLG was not considered a standard practice in matched-related transplants but rather widely recommended. Switching to a different T-cell depletion approach in this setting, mainly using posttransplant cyclophosphamide (PTCy), could possibly improve both GvHD incidence and transplant outcomes, as also suggested by recent experiences (13, 37, 38).

Overall, our data confirmed that full-dose Treo (42 g/m^2^) displays a myeloablative potential associated with a prompt achievement of full donor chimerism and a safe toxicity profile, mostly in the younger population. A lower Treo dose should be adopted for older patients, according to the EMA schedule (30 g/m^2^). This conditioning regimen can be safely adopted as a backbone for newly transplant strategies implementing different GvHD prophylaxis or posttransplant maintenance therapies.

## Data Availability Statement

The raw data supporting the conclusions of this article will be made available by the authors, without undue reservation.

## Ethics Statement

The studies involving human participants were reviewed and approved by San Raffaele’s Hospital Ethics Committee. The patients/participants provided their written informed consent to participate in this study.

## Author Contributions

All authors contributed to patient clinical care and data collection. LL, ML, SM, RG, FC, and JP updated and interpreted the long-term follow-up data. LL and AR performed statistical analysis and prepared the figures and tables. FC and JP designed the study. LL, AR, ML, RG, FC, and JP wrote the manuscript. All authors contributed to the article and approved the submitted version.

## Conflict of Interest

The authors declare that the research was conducted in the absence of any commercial or financial relationships that could be construed as a potential conflict of interest.

## Publisher’s Note

All claims expressed in this article are solely those of the authors and do not necessarily represent those of their affiliated organizations, or those of the publisher, the editors and the reviewers. Any product that may be evaluated in this article, or claim that may be made by its manufacturer, is not guaranteed or endorsed by the publisher.
