# Supplementary material for: Treosulfan-Based Conditioning Regimen Prior to Allogeneic Stem Cell Transplantation: Long-Term Results From a Phase 2 Clinical Trial
Source: Front Oncol. 2021 Sep 10;11:731478. doi: 10.3389/fonc.2021.731478 (PMC8461186; doi:10.3389/fonc.2021.731478)
Supplement: Supplementary file 1 [file DataSheet_1.docx]

**Supplementary material**

**Table S1.** Inclusion and exclusion criteria of “AlloTreo” trial.

| **INCLUSION CRITERIA** |
| --- |
| 1. Patients with haematological malignancies: acute myeloid leukemia (AML), acute lymphoblast leukemia (ALL), chronic myeloid leukemia (CML), myelodysplastic syndrome (MDS), diffuse large B-cell lymphoma (DLBCL), Hodgkin lymphoma (HL), chronic lymphocytic leukemia (CLL), multiple myeloma (MM), or other hematological malignancies with an indication for allogeneic transplantation  2. Availability of an HLA-identical sibling donor (MRD) or HLA-identical unrelated donor (MUD); HLA-identity defined by the following markers: A, B, DRB1, DQB1  3. Target graft size (unmanipulated):  - Bone marrow: 2 - 10 x10^6^ CD34+ cells/Kg BW recipient or >2 x 10^8^ nucleated cells/Kg BW recipient  - Peripheral blood: 4 - 10 x10^6^ CD34+ cells/Kg BW recipient  4. Age > 18 and < 70 years (65 for MUD only)  5. Karnofsky Index > 80 %  6. Adequate contraception in female patients of child-bearing potential  7. Written informed consent |
| **EXCLUSION CRITERIA** |
| 1. Secondary malignancies  2. Previous allogeneic transplantation  3. Severe concomitant illnesses or medical conditions (e.g. impaired respiratory and/or cardiac function)  4. Known and manifested malignant involvement of the CNS  5. Active infectious disease  6. HIV-positivity or active hepatitis infection  7. Impaired liver function (bilirubin > upper normal limit; transaminases >3.0 x upper normal limit)  8. Impaired renal function (creatinine-clearance <60 ml/min; serum creatinine >1.5 x upper normal limit)  9. Pleural effusion or ascites > 1.0 L  10. Pregnancy or lactation  11. Known hypersensitivity to treosulfan and/or fludarabine  12. Participation in another experimental drug trial within 4 weeks before day -6  13. Non-co-operative behavior or non-compliance  14. Psychiatric diseases or conditions that might impair the ability to give informed consent |

**Figure S1.** Treosulfan-based conditioning regimen.

**Figure S2.** Graphic representation of organ involvement of the 25 recipients diagnosed with moderate-to-severe chronic graft-versus-host (cGvHD) subdivided according to donor type. *Patients n°34 and n°57 had also a renal involvement of cGvHD in the form of membranoproliferative glomerulonephritis. GI, gastrointestinal; NIH, National Institute of Health; MRD, matched related donor; MUD, matched unrelated donor; MMUD, 9/10-mismatched unrelated donor.
