# Supplementary figures and images for: Treosulfan-Based Conditioning Regimen Prior to Allogeneic Stem Cell Transplantation: Long-Term Results From a Phase 2 Clinical Trial
Source: Front Oncol. 2021 Sep 10;11:731478. doi: 10.3389/fonc.2021.731478 (PMC8461186; doi:10.3389/fonc.2021.731478)

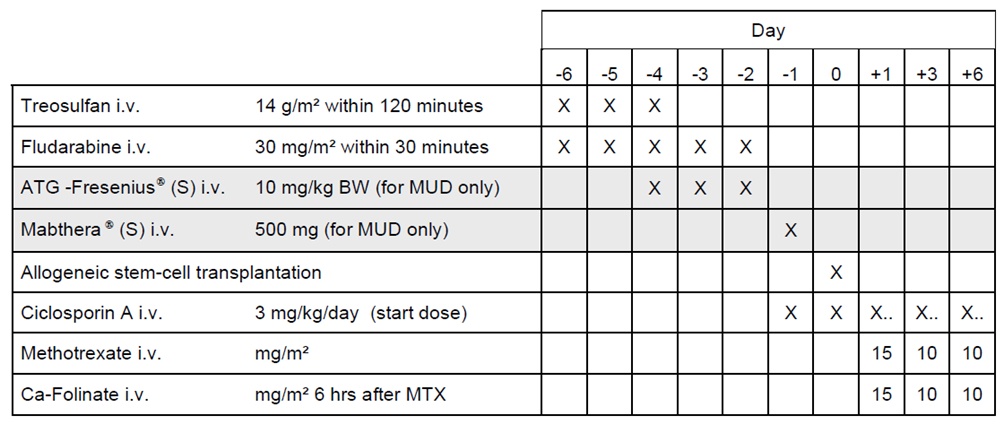

Supplement: Supplementary file 2 [file Image_1.jpeg]

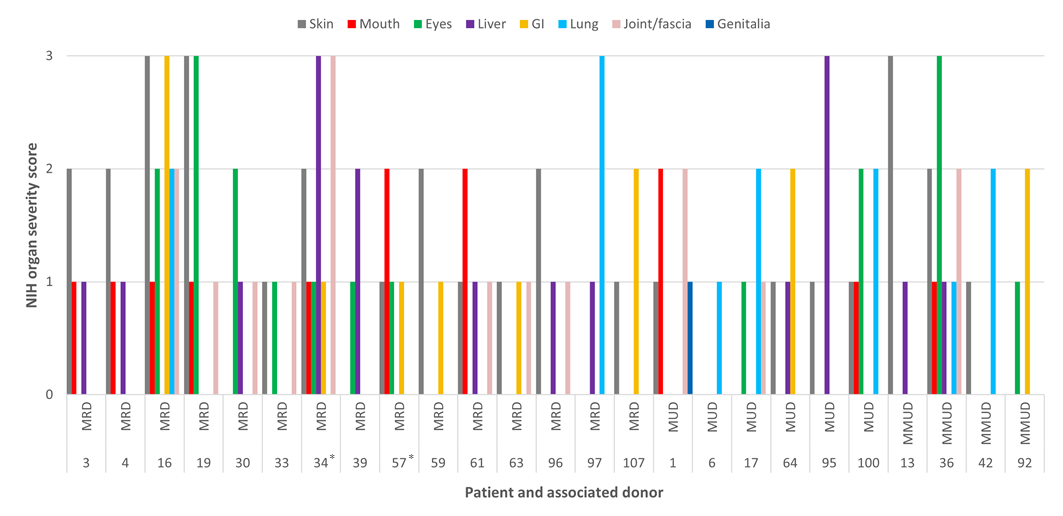

Supplement: Supplementary file 3 [file Image_2.jpeg]
